# Validation with the Dynamic Prediction Model of Protein and Amino Acid Requirements for Growth Performance and Health in Layer Chicks

**DOI:** 10.3390/ani15131968

**Published:** 2025-07-04

**Authors:** Zhi-Yuan Xia, Alainaa Refaie, Miao Liu, You-Yang Wei, Lv-Hui Sun, Zhang-Chao Deng

**Affiliations:** State Key Laboratory of Agricultural Microbiology, Key Laboratory of Smart Farming Technology for Agricultural Animals of Ministry of Agriculture and Rural Affairs, Hubei Hongshan Laboratory, Frontiers Science Center for Animal Breeding and Sustainable Production, College of Animal Science and Technology, Huazhong Agricultural University, Wuhan 430070, China; zhiyuanxia@webmail.hzau.edu.cn (Z.-Y.X.); alainaa.refaie@webmail.hzau.edu.cn (A.R.); lareina.miao@webmail.hzau.edu.cn (M.L.); mmmm1111.vd@webmail.hzau.edu.cn (Y.-Y.W.)

**Keywords:** Jing Tint 6, layer chicks, protein requirements, growth performance, model validation

## Abstract

This study evaluated an established dynamic prediction model for protein and amino acid nutritional requirements on growth performance and health in layer chicks. A total of 288 one-day-old healthy Jing Tint 6 chicks were randomly divided into four treatment groups with 6 replicates of 12 chicks each. The chicks in the four groups were fed a basal diet (BD) according to the feeding standards and a model diet (MD) based on the dynamic prediction model for protein and amino acid values at 90%, 100%, or 110% for 6 weeks. The results showed that, compared to the BD, the 110% MD increased (*p* < 0.05) the FI and the 100% MD had no effect (*p* > 0.05) on FI, BWG, and FCR of chicks throughout the entire feeding phase. The 90% MD, however, significantly reduced (*p* < 0.05) FI and BWG, while also increasing (*p* < 0.05) FCR during the 3–6- and 0–6-week periods. Additionally, compared to the BD, the 100% MD had a lower (*p* < 0.05) intake of crude protein, methionine, lysine, and threonine than that of the BD. Both the 100% and 110% MDs increased (*p* < 0.05) bursa weight and its index at the 2nd week, while the 90% MD reduced (*p* < 0.05) the weights of the liver, spleen, and pancreas at the 6th week when compared to the BD. Moreover, the 100% MD increased (*p* < 0.05) the weights of the duodenum and jejunum, while the 90% MD decreased (*p* < 0.05) jejunum and ileum length at the 2nd or 6th week. Furthermore, no differences (*p* > 0.05) were found in serum ALT, AST, BUN, and UA between the 100% MD and BD groups. In conclusion, the dynamic prediction model can minimize protein waste while supporting healthy growth and development in layer chicks.

## Simple Summary

Precision feeding needs dynamic feed formulations that are produced by adjusting the dietary ingredients based on the specific nutritional requirements of animals. Providing precise nutrition for chickens at different life stages can achieve a balance between their nutritional supply and demand, effectively improving feed utilization and reducing costs. This study validated the dynamic prediction model established for the protein and amino acid nutritional requirements of Jing Tint 6 layer chicks aged 0–6 weeks. The results show that dietary supplementation with a 100% model diet formulated based on the prediction model had no adverse effect growth performance and health status when compared to the basal diet formulated according to the feeding standards for layer chicks. Additionally, the 100% model diet appears to be advantageous for the growth and development of both the bursa and small intestine in these chicks. Moreover, diets formulated based on the prediction model provided more precise nutritional levels of supplemented protein and amino acids, thereby minimizing the waste of these nutrients. These findings indicate that the model effectively predicts the nutritional requirements of Jing Tint 6 layer chicks and holds promise for efficient applications in optimizing feed formulations to enhance economic outcomes within the poultry industry.

## 1. Introduction

The nutritional requirements of livestock and poultry exhibit dynamic changes over time [1]. Each class of animals, depending on their age and growth stage, has specific nutrient requirements that must be met with feeding formulas [2]. For layer chicks, meeting these requirements through precise dietary formulations is critical for promoting organ growth and development during the brooding period, thus improving their performance during the egg-laying period [3,4]. However, conventional feeding standards often exceed the actual nutritional requirements of animals [2]. Consequently, diets formulated using these standards may either exceed or be deficient for the animals’ actual nutritional requirements, leading to inefficiencies in nutrient utilization [5]. An excessive or deficient nutrient supply, particularly in terms of protein and amino acids, can negatively impact growth and production [6]. Therefore, maintaining a balance between nutrient supply and demand is critical for optimizing production performance while ensuring the profitability and sustainability of the poultry industry [7].

Precision feeding, also referred to as individualized nutrition, enables the development of dynamic feed formulations by adjusting dietary ingredients based on an individual’s specific nutritional requirements [8,9,10]. Through precision feeding technology, providing precise nutrition for animals at different life stages can achieve a balance between their nutritional supply and demand, effectively improving feed utilization, reducing costs, and minimizing environmental pollution [11]. Notably, dynamic models such as those from Gompertz [12] and Bridges [13] have been applied to describe growth curves and patterns in various animals. By incorporating mathematical equations, these models can more accurately predict the nutritional requirements, feed intake, and growth rates of animals at different feeding stages, thereby enhancing the precision of dietary formulations. Thus, establishing a dynamic model for nutritional requirements is a key step toward achieving precision feeding.

In a previous study, we established a dynamic prediction model for the protein and amino acid nutritional requirements of Jing Tint 6 chicks [14]. However, any model must be validated and evaluated to ensure its practical applicability, as predicting its effectiveness across all conditions is challenging [15,16]. Therefore, this study aims to evaluate the accuracy of our established model by comparing the growth performance and health of layer chicks fed diets based on the model’s predicted values against those formulated using conventional feeding standards. This will help facilitate the application of dynamic models in poultry production, contributing to more efficient and sustainable practices in the industry.

## 2. Materials and Methods

### 2.1. Birds, Diets, and Treatments

The animal experimental protocol was approved by the Institutional Animal Care and Use Committee of Huazhong Agricultural University, China (ethical code: HZAUCH-2024-0006). A total of 288 one-day-old healthy Jing Tint 6 chicks (33.44 ± 0.08 g) were randomly divided into four treatment groups with 6 replicates of 12 chicks/cage. Each group of 12 chicks was housed in a separate cage (0.8 m × 0.8 m × 0.5 m) as one replicate. The density of each group was the same during the feeding phase. The chicks from the four groups were fed either a basal diet (BD), formulated according to the feeding standards for Jing Tint 6 chicks, or the model diet (MD), formulated according to the dynamic prediction model for protein and amino acid values at 90%, 100%, or 110% for 6 weeks. More details about the dynamic prediction model can be found in our previously published study [14]. The composition and nutritional values of the diets are shown in Table 1. Suitable indoor temperature and humidity, good ventilation, and consistent feeding and management conditions were maintained across all groups [17,18]. The chicks were fed for a period of 6 weeks, during which they were provided with feed and water ad libitum. The body weight (BW) and feed intake (FI) were recorded weekly for each cage (total 6 cages). The body weight gain (BWG) and feed conversion ratio (FCR) were calculated based on BW and FI. BEG (g) = final BW (g) − initial BW (g); FCR = FI (g)/BWG (g). The protein and main amino acid intakes of chicks during the 0–6-week period were calculated based on the FI and nutritional levels of the diet. The calculation formula is as follows: protein and amino acid intake (g) = FI (g) × protein and amino acid levels in the diet (%).

### 2.2. Sample Collection and Measurement

At the end of 2nd and 6th weeks, two chicks with average BW for each cage were selected. After a 12 h fasting period, 12 chicks from each group were euthanized for sampling and measuring. The vertical distance from the top of the right appendage joint to the bottom of the claws of the chick was measured as the tibial length statistics. Lay the chick on its back with the sternum facing upwards. Press it away from the crop to expose the anterior opening of the chest. Insert the syringe needle along the depression angle of the collarbone, move it horizontally along the midline of the body, and insert it into the heart to collect blood. Serum was subsequently collected from heart blood and then stored at −20 °C for further analysis [19,20]. Subsequently, the liver, spleen, pancreas, and bursae were removed completely and weighed. The organ weight indexes were calculated based on the below formula [21,22]: organ index (%) = organ weight (g)/body weight (g) × 100. Meanwhile, the weight and length of the duodenum, jejunum, and ileum were also measured.

### 2.3. Serum Biochemistry Analyses

The serum parameters, including alanine aminotransferase (ALT), aspartate aminotransferase (AST), uric acid (UA), and urea nitrogen (BUN) were determined by the corresponding test kits (C009-2-1, C010-2-1, C012-1-1, and C013-2-1; Nanjing Jiancheng Bioengineering Institute; Nanjing, China) according to the manufacturer’s instructions, as previously described [23]. All 12 serum samples were tested.

### 2.4. Statistical Analyses

The results are presented as the mean ± standard deviation (SD). Statistical analyses were carried out using SPSS 26.0 software. One-way analysis of variance (ANOVA) was used to analyze all the collected data, followed by Duncan’s multiple comparison test. Significant differences were calculated at the *p* < 0.05 level for all statistical analyses.

## 3. Results

### 3.1. Growth Performance

The growth performance results are shown in Table 2. Compared to the BD, the 110% MD exhibited a significant increase (*p* < 0.05) in FI throughout the entire feeding phase, while BWG and FCR remained unaffected (*p* > 0.05). Notably, the 100% MD had no effect (*p* > 0.05) on the FI, BWG, and FCR of chicks compared to the BD throughout the entire feeding phase. However, compared to the BD, the 100% MD, and the 110% MD groups, the 90% MD significantly reduced (*p* < 0.05) FI and BWG, as well as increasing (*p* < 0.05) the FCR during weeks 3–6 and weeks 0–6.

### 3.2. Protein and Main Amino Acid Intake

The results for the protein and main amino acid intake are shown in Table 3. Compared to the BD, the 110% MD significantly increased (*p* < 0.05) the chicks’ intake of crude protein, lysine, and threonine during weeks 0–6 by 13.8%, 15.0%, and 6.5%, respectively, while reducing the methionine intake by 33.8%. However, compared to the BD and 110% MD groups, dietary supplementation with both the 90% and 100% MDs significantly reduced (*p* < 0.05) the chicks’ intake of crude protein, methionine, lysine, and threonine during weeks 0–6. Compared to the BD, the 100% MD significantly reduced (*p* < 0.05) the chicks’ intake of crude protein, methionine, lysine, and threonine by 5.8%, 45.1%, 9.4%, and 11.9%, respectively.

### 3.3. Organ Weight and Their Indexes

**Figure 1:**
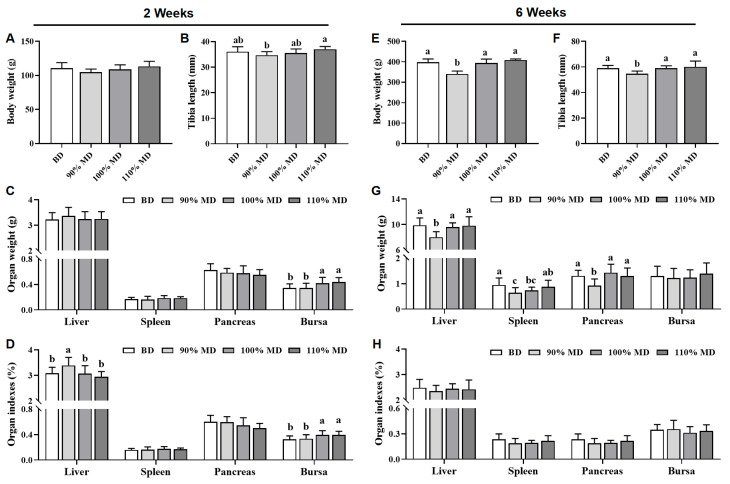
Effect of model diet on body weight (**A**,**E**), tibia length (**B**,**F**), main organ weight (**C**,**G**), and organ indexes (**D**,**H**) of Jing Tint 6 chicks at the 2nd and 6th weeks. Values are means ± SD, *n* = 12. Labeled means in a row with different letters differ significantly, *p* < 0.05. BD, basal diet; MD, model diet.

Compared to the BD, both the 100% and 110% MDs had no effect (*p* > 0.05) on the body weight and tibia length of chicks at 2nd and 6th weeks, while the 90% MD significantly reduced (*p* < 0.05) the body weight and tibia length at 6th week (Figure 1A,B,E,F). At the 2nd week, both the 100% and 110% MDs increased (*p* < 0.05) bursa weight and its index, and the 90% MD increased the liver index (Figure 1C,D). At the 6th week, the 90% MD reduced (*p* < 0.05) the weights of the liver, spleen, and pancreas when compared to the BD (Figure 1G). However, no differences (*p* > 0.05) were found in the main organ indexes among the four groups at the 6th week (Figure 1H).

### 3.4. Small Intestine Length and Weight

**Figure 2:**
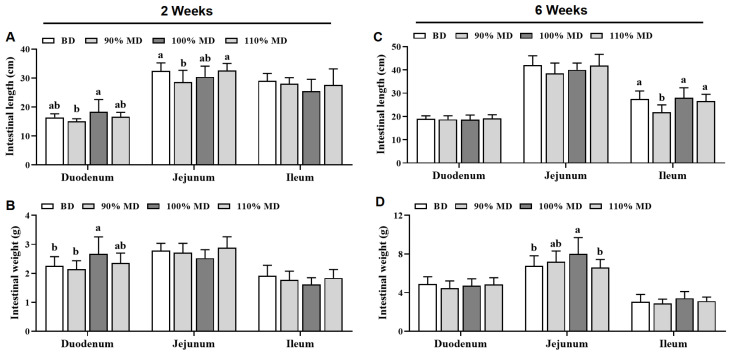
Effect of model diet on the small intestine length (**A**,**C**) and weight (**B**,**D**) in Jing Tint 6 chicks at the 2nd and 6th weeks. Values are means ± SD, *n* = 12. Labeled means in a row with different letters differ significantly, *p* < 0.05. BD, basal diet; MD, model diet.

The lengths and weights of the chicks’ small intestines are shown in Figure 2. At the 2nd week, compared to the BD, the 100% MD increased (*p* < 0.05) duodenum weight, while the 90% MD decreased jejunum length (Figure 2A,B). At the 6th week, compared to the BD, the 100% MD increased (*p* < 0.05) jejunum weight, while the 90% MD decreased ileum length (Figure 2C,D). Notably, no differences (*p* > 0.05) were found in the length and weight of parts of the small intestine, including the duodenum, jejunum, and ileum, between the BD and the 110% MD at the 2nd and 6th weeks (Figure 2).

### 3.5. Serum Biochemistry

**Figure 3:**
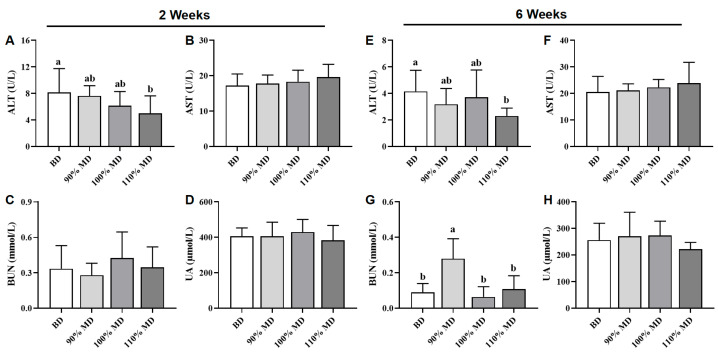
Effect of model diet on serum ALT (**A**,**E**), AST (**B**,**F**), BUN (**C**,**G**), and UA (**D**,**H**) in Jing Tint 6 chicks at the 2nd and 6th weeks. Values are means ± SD, *n* = 12. Labeled means in a row with different letters differ significantly, *p* < 0.05. ALT, alanine aminotransferase; AST, aspartate aminotransferase; BUN, urea nitrogen; UA, uric acid; BD, basal diet; MD, model diet.

The serum biochemistry results are shown in Figure 3. At the 2nd week, compared to the BD, the 110% MD reduced (*p* < 0.05) serum ALT, while no differences (*p* > 0.05) were found in serum AST, BUN, and UA among the four groups (Figure 3A–D). At the 6th week, compared to the BD, the 110% MD also reduced (*p* < 0.05) serum ALT; the 90% MD increased serum BUN, while no differences (*p* > 0.05) were found in serum AST and UA among the four groups (Figure 1E–H).

## 4. Discussion

Precision feeding needs dynamic feed formulations that are produced by adjusting the dietary ingredients based on the specific nutritional requirements of animals [9]. Thus, accurately estimating an animal’s nutritional requirements via establishing the optimal dynamic model is vital for precision feeding [24,25]. In our previous study [14], a dynamic prediction model of the protein and amino acid requirements of Jing Tint 6 chicks during the brooding period was established. In this study, the dynamic model was applied to calculate the weekly protein and amino acid requirements of chicks, which guided the preparation of dietary formulations to validate the model diet’s effect on the growth performance and health of layer chicks. Growth performance serves as a crucial indicator for assessing the growth and development of laying hens during the brooding period [26]. The current study shows that dietary supplementation with 100% and 110% MDs had no adverse effect on the growth performance of chicks compare with the BD throughout the entire feeding phase. However, the 90% MD significantly reduced the growth performance of chicks, as evidenced by the decreasing BW, FI, and BWG, as well as the increasing FCR during weeks 3–6. These outcomes suggesting that insufficient nutrient supply can lead to growth retardation [27] and that the feed prepared according to our predicted dynamic model can meet the nutritional requirements for the normal growth of chicks.

During brooding period, the layer chicks exhibit vigorous growth and metabolism and high dietary requirements for protein and amino acids [28,29,30]. However, high protein levels can lead to metabolic stress, feed resource waste, and environmental pollution [31]. Conversely, low protein levels may fail to meet the growth requirements of chicks, hindering their development and affecting later egg-laying performance [32]. Previous studies have demonstrated that increasing or decreasing the dietary levels of protein and amino acids had a significant impact on the tibia development of laying hens [33,34]. Thus, tibia length is the primary criteria for evaluation of whether dietary protein and amino acids levels meet the development requirements of layer chicks. In this study, the tibia length of laying chicks was not changed by the 100% and 110% MD treatments, indicating that the protein and amino acid levels predicted by the model could meet the growth requirements of laying chicks during the brooding stages. Notably, compared to the BD, dietary supplementation with the 100% MD significantly reduced the chicks intake of crude protein, methionine, lysine, and threonine by 5.8–45.1%. These results suggest that the feed prepared according to our predicted dynamic model could reduce nitrogen intake without any negative effects on the growth and development of laying chicks, which could mean that lower amounts of protein feed ingredient are required, which could lead to economic benefits.

The indices of an animal’s main organs can be used as an indicator to reflect their function [35]. Within a certain range, a higher immune organ index correlates with better-developed immune organs and a stronger ability to resist diseases [36]. The bursa, a primary lymphoid organ responsible for the maturation of B lymphocytes in birds, plays a crucial role in antibody production [37,38]. In this study, dietary supplementation with the 100% and 110% MDs showed a significant increase in the bursa index of the laying chicks at the 2nd week. These findings suggest that the MDs made based on our constructed dynamic model have a positive impact on the development of the bursa, which highlights the importance of precision nutrition in enhancing the immune system and overall health of laying chicks.

The gastrointestinal tract plays an important role in digestion and nutrient absorption [39,40]; intestinal development directly affects the digestion, absorption, and utilization of feed nutrients [41]. In this study, the weights of the duodenum and jejunum of chicks fed the 100% MD were significantly heavier than those of chicks fed the BD at the 2nd and 6th weeks, respectively. This result indicates that the nutritional requirements predicted by the dynamic model can promote the early development of small intestine in layer chicks. Notably, dietary supplementation with the 90% MD decreased the length of the jejunum and ileum, suggesting that nutritional supply below the model-predicted values could not meet the growth requirements of the small intestine in layer chicks.

Protein is an important nutrient in poultry feed and its metabolism plays a vital role in maintaining the normal host physiological function [42,43,44]. BUN, a by-product of protein metabolism, can reflect the level of protein breakdown in the body [45]. In this study, the BUN level in laying chicks was significantly increased under the 90% MD treatment, indicating that protein metabolism is impaired when the nutritional supply of the feed does not meet the predicted level of the model. AST and ALT, the two most active transaminases in the body, play an important role in amino acid metabolism [46,47,48]. In this study, the 110% MD showed a significant decrease in serum ALT levels, indicating a deterioration in protein synthesis. Notably, dietary supplementation with the 100% MD had no adverse effect on the serum biochemistry, as no differences were found in the serum ALT, AST, BUN, and UA between the BD and 100% MD groups. Taken together, these outcomes suggest that increasing or decreasing the dietary levels of protein and amino acids has an adverse effect on the physiological metabolism of chicks, highlighting the importance of nutritional levels for maintaining physiological health in chicks during the brooding period.

## 5. Conclusions

This study validated the established dynamic prediction model for the protein and amino acid nutritional requirements of Jing Tint 6 layer chicks. Dietary supplementation with the 100% MD resulted in similar growth performance and health status compared to the BD. Meanwhile, the 100% MD could beneficial for the growth and development of the bursae and small intestines of chicks. Moreover, diets formulated according to the prediction model provided more accurate required nutritional levels for supplemented protein and amino acids, thus reducing the waste of these nutrients. These findings indicate that the model accurately predicts the nutritional requirements of Jing Tint 6 layer chicks and has potential efficiency applications in optimizing feed formulations for economic improvements in the poultry industry.

## Figures and Tables

**Table 1:** Composition and nutritional values of the diet.

Component, %	Basal Diet (BD)		Model Diet (MD)																	
	1–2 Week	3–6 Week	1 Week			2 Week			3 Week			4 Week			5 Week			6 Week		
			90%	100%	110%	90%	100%	110%	90%	100%	110%	90%	100%	110%	90%	100%	110%	90%	100%	110%
Corn	63.80	66.64	65.77	59.04	53.22	67.93	61.25	54.25	73.89	68.30	62.52	72.64	66.84	60.60	75.12	69.93	64.37	78.35	73.73	68.74
Soybean meal	31.37	28.60	28.85	34.88	40.72	27.21	33.09	39.04	21.47	26.63	31.82	22.73	28.03	33.44	20.29	25.26	30.32	17.09	21.67	26.32
CaCo_3_	1.16	1.43	1.75	1.70	1.10	1.63	1.60	1.66	1.85	1.77	1.66	1.85	1.735	1.72	1.85	1.81	1.71	1.85	1.84	1.76
Calcium hydrogen phosphate	1.90	1.86	1.90	1.90	1.86	1.92	1.90	1.90	1.87	1.86	1.87	1.89	1.86	1.87	1.89	1.86	1.88	1.91	1.92	1.90
Soybean oil	0.80	0.50	0.80	1.50	2.08	0.40	1.20	2.15	-	0.45	1.10	-	0.60	1.40	-	0.25	0.80	-	-	0.40
Salt	0.42	0.43	0.42	0.42	0.42	0.42	0.42	0.42	0.42	0.43	0.42	0.42	0.43	0.42	0.43	0.43	0.42	0.43	0.43	0.42
Premix ^1^	0.07	0.07	0.07	0.07	0.07	0.07	0.07	0.07	0.07	0.07	0.07	0.07	0.07	0.07	0.07	0.07	0.07	0.07	0.07	0.07
50% choline chloride	0.10	0.10	0.10	0.10	0.10	0.10	0.10	0.10	0.10	0.10	0.10	0.10	0.10	0.10	0.10	0.10	0.10	0.10	0.10	0.10
L-lysine	0.04	0.015	-	-	-	-	-	-	-	-	-	-	-	-	-	-	-	-	-	-
DL Methionine	0.21	0.21	-	-	-	-	-	-	-	-	-	-	-	-	-	0.01	0.01	0.04	0.045	0.055
L-valine	-	-	0.27	0.32	0.37	0.25	0.30	0.35	0.23	0.28	0.33	0.20	0.245	0.29	0.15	0.19	0.23	0.07	0.10	0.14
L-threonine	0.13	0.145	0.07	0.07	0.06	0.07	0.07	0.06	0.10	0.11	0.11	0.10	0.09	0.09	0.10	0.09	0.09	0.09	0.095	0.095
Total	100	100	100	100	100	100	100	100	100	100	100	100	100	100	100	100	100	100	100	100
**Nutritional level ^2^**																				
Metabolic energy (Mcal/kg)	2.92	2.92	2.92	2.92	2.92	2.92	2.92	2.92	2.92	2.92	2.92	2.92	2.92	2.92	2.92	2.92	2.92	2.92	2.92	2.92
Crude protein	20.00	19.00	19.03	21.15	23.26	18.49	20.54	22.60	16.43	18.26	20.09	16.89	18.77	20.64	16.01	17.79	19.57	14.86	16.51	18.16
Calcium	0.98	1.06	1.18	1.18	0.98	1.14	1.14	1.18	1.20	1.18	1.15	1.20	1.17	1.18	1.20	1.19	1.17	1.19	1.20	1.18
Total phosphorus	0.46	0.45	0.46	0.46	0.46	0.46	0.46	0.46	0.45	0.45	0.45	0.45	0.45	0.45	0.45	0.45	0.45	0.45	0.45	0.45
Effective phosphorus	0.46	0.45	0.46	0.46	0.46	0.46	0.46	0.46	0.45	0.45	0.45	0.45	0.45	0.45	0.45	0.45	0.45	0.45	0.45	0.45
Methionine	0.46	0.45	0.25	0.27	0.29	0.24	0.26	0.29	0.22	0.24	0.26	0.22	0.24	0.27	0.21	0.24	0.26	0.24	0.26	0.29
Met + Cys	0.92	0.89	0.69	0.75	0.82	0.67	0.73	0.80	0.60	0.66	0.72	0.62	0.68	0.74	0.59	0.66	0.71	0.59	0.65	0.71
Lysine	1.10	1.00	0.99	1.16	1.32	0.95	1.11	1.27	0.79	0.93	1.08	0.83	0.97	1.12	0.76	0.90	1.04	0.67	0.80	0.93
Threonine	0.75	0.73	0.66	0.74	0.81	0.64	0.72	0.79	0.60	0.67	0.74	0.61	0.67	0.74	0.58	0.64	0.70	0.53	0.59	0.65
Isoleucine	0.98	0.91	0.92	1.06	1.20	0.88	1.02	1.16	0.74	0.87	0.99	0.77	0.90	1.03	0.71	0.83	0.95	0.64	0.75	0.86
Valine	0.76	0.73	0.99	1.10	1.21	0.96	1.06	1.17	0.89	0.98	1.08	0.87	0.96	1.06	0.79	0.88	0.97	0.69	0.76	0.84

^1^ Vitamin and mineral premix provided/kg diet: iron, 100 mg; copper, 8 mg; manganese, 20 mg; zinc, 100 mg; selenium, 0.3 mg; iodine, 0.7 mg; retinyl acetate, 10,280 IU; cholecalciferol 2280 IU; dl-a-tocopheryl acetate,17.12 mg; menadione, 6.82 mg; thiamin, 2.28 mg; riboflavin, 5.68 mg; pantothenic acid, 12.25 mg; pyridoxine, 2.28 mg; niacin, 22.84 mg; biotin, 0.18 mg; folic acid, 1.12 mg. ^2^ Calculated.

**Table 2:** Effect of model diet on growth performance of Jing Tint 6 chicks ^1^.

	BD	90% MD	100% MD	110% MD	*p*-Value
0–2 Weeks					
FI (g/chick)	143.46 ± 3.28 ^b^	143.45 ± 3.89 ^b^	144.13 ± 6.16 ^b^	156.47 ± 4.65 ^a^	0.0001
BWG (g/chick)	77.26 ± 8.12	71.46 ± 4.40	75.54 ± 6.46	79.81 ± 7.59	0.2212
FCR	1.87 ± 0.18	2.01 ± 0.11	1.91 ± 0.10	1.97 ± 0.17	0.3797
3–6 Weeks					
FI (g/chick)	696.45 ± 22.75 ^b^	652.71 ± 22.63 ^c^	689.26 ± 16.47 ^b^	758.27 ± 24.20 ^a^	0.0001
BWG (g/chick)	287.72 ± 22.05 ^a^	235.74 ± 9.89 ^b^	285.09 ± 21.13 ^a^	295.54 ± 6.93 ^a^	0.0001
FCR	2.43 ± 0.22 ^b^	2.77 ± 0.14 ^a^	2.43 ± 0.16 ^b^	2.57 ± 0.10 ^ab^	0.0047
0–6 Weeks					
FI (g/chick)	839.90 ± 24.89 ^b^	796.15 ± 20.39 ^c^	833.39 ± 11.53 ^b^	914.75 ± 23.52 ^a^	0.0001
BWG (g/chick)	364.98 ± 15.50 ^a^	307.20 ± 13.93 ^b^	360.63 ± 19.28 ^a^	375.35 ± 5.19 ^a^	0.0001
FCR	2.31 ± 0.13 ^b^	2.60 ± 0.12 ^a^	2.32 ± 0.12 ^b^	2.44 ± 0.05 ^b^	0.0005

^1^ Values are means ± SD, *n* = 6. Labeled means in a row with different letters differ significantly, *p* < 0.05. FI, Feed intake; BWG, Body weight gain; FCR, Feed conversion ratio; BD, basal diet; MD, model diet.

**Table 3:** Effect of model diet on protein and main amino acids intake of Jing Tint 6 chicks during weeks 0–6 ^1^.

	BD	90% MD	100% MD	110% MD	*p*-Value
Crude protein intake (g/chick)	161.02 ± 4.75 ^b^	130.56 ± 3.23 ^d^	151.68 ± 1.99 ^c^	183.18 ± 4.70 ^a^	0.0001
Methionine intake (g/chick)	3.79 ± 0.11 ^a^	1.81 ± 0.05 ^d^	2.08 ± 0.03 ^c^	2.51 ± 0.06 ^b^	0.0001
Lysine intake (g/chick)	8.54 ± 0.25 ^b^	6.28 ± 0.15 ^d^	7.74 ± 0.10 ^c^	9.82 ± 0.25 ^a^	0.0001
Threonine intake (g/chick)	6.16 ± 0.18 ^b^	4.67 ± 0.12 ^d^	5.43 ± 0.07 ^c^	6.56 ± 0.17 ^a^	0.0001

^1^ Values are means ± SD, *n* = 6. Labeled means in a row with different letters differ significantly, *p* < 0.05. BD, basal diet; MD, model diet.

## Data Availability

The original contributions presented in the study are included in the article.

## References

[1] Caldas J.V., Boonsinchai N., Wang J.R., England J.A., Coon C.N. (2019). The dynamics of body composition and body energy content in broilers. Poult. Sci..

[2] Tian Y.D. (2005). Dynamie Model Construction of Energy and Amino Acid Requirements for Broilers. Ph.D. Thesis.

[3] Alfarisy G.A.F., Mahmudy W.F., Natsir M.H. (2018). Good parameters for PSO in optimizing laying hen diet. Int. J. Electr. Comput. Eng..

[4] Li G., Feng Y., Cui J., Hou Q., Li T., Jia M., Lv Z., Jiang Q., Wang Y., Zhang M. (2023). The ionome and proteome landscape of aging in laying hens and relation to egg white quality. Sci. China Life Sci..

[5] Pomar C., Hauschild L., Zhang G.-H., Pomar J., Lovatto P.A. (2009). Applying precision feeding techniques in growing-finishing pig operations. Rev. Bras. Zootec..

[6] Fan L., Xia Y., Wang Y., Han D., Liu Y., Li J., Fu J., Wang L., Gan Z., Liu B. (2023). Gut microbiota bridges dietary nutrients and host immunity. Sci. China Life Sci..

[7] Bamiro O.M., Shittu A.M. (2009). Vertical integration and cost behavior in poultry industry in Ogun and Oyo States of Nigeria. Agribusiness.

[8] Cao K.X., Deng Z.C., Li S.J., Yi D., He X., Yang X.J., Guo Y.M., Sun L.H. (2024). Poultry nutrition: Achievement, challenge, and strategy. J. Nutr..

[9] Zuidhof M.J., Fedorak M.V., Ouellette C.A., Wenger I.I. (2017). Precision feeding: Innovative management of broiler breeder feed intake and flock uniformity. Poult. Sci..

[10] Li Z., Li Y., Lv Z., Liu H., Zhao J., Noblet J., Wang F., Lai C., Li D. (2017). Net energy of corn, soybean meal and rapeseed meal in growing pigs. J. Anim. Sci. Biotechnol..

[11] Pawlowska J., Sosnówka-Czajka E. (2019). Factors affecting chick quality in Poland. Worlds Poult. Sci. J..

[12] Gompertz B. (1815). On the nature of the function expressive of the law of human mortality, and on a new mode of determining the value of life contingencies. Proc. R. Soc. Lond..

[13] Bridges T.C., Turner L.W., Stahly T.S., Usry J.L., Loewer O.J. (1992). Modeling the physiological growth of swine part i: Model logic and growth concepts. Trans. ASAE.

[14] Liu M., Xia Z.Y., Li H.L., Huang Y.X., Refaie A., Deng Z.C., Sun L.H. (2024). Estimation of protein and amino acid requirements in layer chicks depending on dynamic model. Animals.

[15] Hurwitz S., Plavnik I., Bartov I., Bornstein S. (1980). The amino acid requirements of chicks: Experimental validation of model-calculated requirements. Poult. Sci..

[16] Hurwitz S., Plavnik I., Bengal I., Talpaz H., Bartov I. (1983). The amino acid requirements of growing turkeys. Experimental validation of model-calculated requirements for sulfur amino acids and lysine. Poult. Sci..

[17] Yan Y.Q., Liu M., Xu Z.J., Xu Z.J., Huang Y.X., Li X.M., Chen C.J., Zuo G., Yang J.C., Lei X.G. (2024). Optimum doses and forms of selenium maintaining reproductive health via regulating homeostasis of gut microbiota and testicular redox, inflammation, cell proliferation, and apoptosis in roosters. J. Nutr..

[18] Yang J.C., Huang Y.X., Sun H., Liu M., Zhao L., Sun L.H. (2023). Selenium deficiency dysregulates one-carbon metabolism in nutritional muscular dystrophy of chicks. J. Nutr..

[19] Huang W., Ma T., Liu Y., Kwok L.Y., Li Y., Jin H., Zhao F., Shen X., Shi X., Sun Z. (2023). Spraying compound probiotics improves growth performance and immunity and modulates gut microbiota and blood metabolites of suckling piglets. Sci. China Life Sci..

[20] Zhao L., Chu X.H., Liu S., Li R., Zhu Y.F., Li F.N., Jiang J., Zhou J.C., Lei X.G., Sun L.H. (2022). Selenium-enriched cardamine violifolia increases selenium and decreases cholesterol concentrations in liver and pectoral muscle of broilers. J. Nutr..

[21] Zhao L., Liu M., Sun H., Yang J.C., Huang Y.X., Huang J.Q., Lei X., Sun L.H. (2023). Selenium deficiency-induced multiple tissue damage with dysregulation of immune and redox homeostasis in broiler chicks under heat stress. Sci. China Life Sci..

[22] Li J., Bai G., Gao Y., Gao Q., Zhong R., Chen L., Wang Y., Ma T., Zhang H. (2024). Solid-state fermentation pro-enzymes supplementation benefits growth performance, health, and intestinal microbiota of broiler chickens fed wheat-based diet. Anim. Res. One Health.

[23] Xu K.L., Gong G.X., Liu M., Yang L., Xu Z.J., Gao S., Xiao M.Y., Ren T., Zhao B.J., Khalil M.M. (2022). Keratinase improves the growth performance, meat quality and redox status of broiler chickens fed a diet containing feather meal. Poult. Sci..

[24] Forester S.M., Jennings-Dobbs E.M., Sathar S.A., Layman D.K. (2023). Perspective: Developing a nutrient-based framework for protein quality. J. Nutr..

[25] Usigbe M.J., Uyeh D.D., Park T., Ha Y., Mallipeddi R. (2025). Many objective optimization and decision support for dairy cattle feed formulation. Sci. Rep..

[26] Narinç D., Aydemir E. (2021). Chick quality: An overview of measurement techniques and influencing factors. Worlds Poult. Sci. J..

[27] Gous R.M. (2010). Nutritional limitations on growth and development in poultry. Livest. Sci..

[28] Andrews T.L., Harms R.H., Wilson H.R. (1973). Protein requirement of the bobwhite chick. Poult. Sci..

[29] Macelline S.P., Toghyani M., Chrystal P.V., Selle P.H., Liu S.Y. (2021). Amino acid requirements for laying hens: A comprehensive review. Poult. Sci..

[30] Macelline S.P., Chrystal P.V., Liu S.Y., Selle P.H. (2021). The dynamic conversion of dietary protein and amino acids into chicken-meat protein. Animals.

[31] Wang J., Yue H., Wu S., Zhang H., Qi G. (2017). Nutritional modulation of health, egg quality and environmental pollution of the layers. Anim. Nutr..

[32] Lesuisse J., Li C., Schallier S., Leblois J., Everaert N., Buyse J. (2017). Feeding broiler breeders a reduced balanced protein diet during the rearing and laying period impairs reproductive performance but enhances broiler offspring performance. Poult. Sci..

[33] Castro F.L.S., Kim H.Y., Hong Y.G., Kim W.K. (2019). The effect of total sulfur amino acid levels on growth performance, egg quality, and bone metabolism in laying hens subjected to high environmental temperature. Poult. Sci..

[34] Dao H.T., Moss A.F., Bradbury E.J., Swick R.A. (2023). Effects of L-arginine, guanidinoacetic acid and L-citrulline supplementation in reduced-protein diets on bone morphology and mineralization of laying hens. Anim. Nutr..

[35] Fu G., Zhang M., Huang Y., Han R., Qi K., Yin L., Zhao D., Huang Y., Ma T., Wang L. (2024). Effects of different addition levels of CHM-JM113 on growth performance, antioxidant capacity, organ index, and intestinal health of AA broilers. Front. Vet. Sci..

[36] Madej J.P., Stefaniak T., Bednarczyk M. (2015). Effect of in ovo-delivered prebiotics and synbiotics on lymphoid-organs’ morphology in chickens. Poult. Sci..

[37] Tsuji T., Miyoshi M. (2001). A Scanning and transmission electron microscopic study of the lymphoreticular framework in the chicken fabricius’ bursa. Med. Bull. Fukuoka Univ..

[38] Cheng J., Lei H., Xie C., Chen J., Yi X., Zhao F., Yuan Y., Chen P., He J., Luo C. (2023). Lymphocyte development in the bursa of fabricius of young broilers is influenced by the gut microbiota. Microbiol. Spectr..

[39] Chukwudi P., Umeugokwe P.I., Ikeh N.E., Amaefule B.C. (2025). The effects of organic acids on broiler chicken nutrition: A review. Anim. Res. One Health.

[40] Deng Z.C., Wang J., Wang J., Yan Y.Q., Huang Y.X., Chen C.Q., Sun L.H., Liu M. (2024). Tannic acid extracted from gallnut improves intestinal health with regulation of redox homeostasis and gut microbiota of weaned piglets. Anim. Res. One Health.

[41] Ravindran V., Abdollahi M.R. (2021). Nutrition and digestive physiology of the broiler chick: State of the art and outlook. Animals.

[42] Beski S.S.M., Swick R.A., Iji P.A. (2015). Specialized protein products in broiler chicken nutrition: A review. Anim. Nutr..

[43] Qaid M.M., Al-Garadi M.A. (2021). Protein and amino acid metabolism in poultry during and after heat stress: A review. Animals.

[44] Liu C., Ma N., Feng Y., Zhou M., Li H., Zhang X., Ma X. (2023). From probiotics to postbiotics: Concepts and applications. Anim. Res. One Health.

[45] Marín-García P.J., Llobat L., López-Lujan M.C., Cambra-López M., Blas E., Pascual J.J. (2022). Urea nitrogen metabolite can contribute to implementing the ideal protein concept in monogastric animals. Animals.

[46] Jegatheesan P., Beutheu S., Ventura G., Sarfati G., Nubret E., Kapel N., Waligora-Dupriet A.J., Bergheim I., Cynober L., De-Bandt J.P. (2016). Effect of specific amino acids on hepatic lipid metabolism in fructose-induced non-alcoholic fatty liver disease. Clin. Nutr..

[47] Deng J., Peng Z., Xia Z.Y., Mo Y.X., Guo L.J., Wei J.T., Sun L.H., Liu M. (2025). Five glutathione S-transferase isozymes played crucial role in the detoxification of aflatoxin B1 in chicken liver. J. Anim. Sci. Biotechnol..

[48] Sun X., Ma J., Wang C., Ren Z., Yang X., Yang X., Liu Y. (2025). Functional roles of folic acid in alleviating dexamethasone-induced fatty liver syndrome in laying hens. Anim. Res. One Health.

